# External Validation of the 4C (Coronavirus Clinical Characterization Consortium) Mortality Score in a Teaching Hospital in Brazil

**DOI:** 10.7759/cureus.76811

**Published:** 2025-01-02

**Authors:** Karima E Bruno, Henrique Mussi, Amanda E Bruno, Juliana B Rodrigues, Manuella Rezende, Victor C Cortes, Ronaldo A Gismondi

**Affiliations:** 1 Medicina Clinica, Hospital Universitário Antônio Pedro (Ebserh/Universidade Federal Fluminense), Niteroi, BRA

**Keywords:** covid-19, covid prognosis, critical care and hospital medicine, in hospital mortality, scores

## Abstract

Background

The 4C (Coronavirus Clinical Characterization Consortium) Mortality Score has demonstrated good discrimination in COVID-19 but has not been widely validated in Brazil. The 4C Mortality Score is a clinical tool developed during the COVID-19 pandemic to predict in-hospital mortality for patients admitted with COVID-19. It was derived from a large dataset of hospitalized patients in the United Kingdom and provides a simple yet effective way to stratify patients based on their risk of death.

Objective

This study aimed to determine the accuracy of the 4C Mortality Score in patients admitted with COVID-19 in a university teaching hospital.

Methods

The study was observational, longitudinal, and retrospective, conducted in a 180-bed university teaching hospital in Rio de Janeiro, Brazil. We included all patients admitted with COVID-19 and followed them until discharge. The 4C Mortality Score was calculated based on age, sex, Charlson index, respiratory rate, peripheral oxygen saturation (room air), Glasgow Coma Scale, serum urea, and C-reactive protein (CRP) level. The primary outcome was mortality.

Results

We included 208 participants, with a median age of 63 years. Among them, 111 (53%) were male; 52 (25%) had cardiovascular disease, and 83 (39%) had cancer. Mortality was 39.9%. Independent predictors of mortality were age, hemoglobin, CRP, mechanical ventilation, and the need for vasopressors. The 4C Mortality Score's area under the receiver operating characteristic curve (AUC-ROC) was 89.9%.

Conclusion

The 4C Mortality Score demonstrated excellent discrimination in a teaching hospital population.

## Introduction

The COVID-19 pandemic has posed an unprecedented global health challenge since its emergence in late 2019. Despite substantial advancements in vaccine development, the continuous emergence of new SARS-CoV-2 variants, the variable immunogenicity of vaccines across different populations, and the rise of anti-vaccination movements have perpetuated the pandemic's burden. As of 2024, the pandemic has caused millions of deaths worldwide, with significant disparities in morbidity and mortality observed across regions. Understanding the disease's clinical course and identifying risk factors for severe outcomes remain paramount to improving patient management and effectively allocating healthcare resources.

One of the most critical aspects of COVID-19 care is predicting patient outcomes, particularly mortality, to guide clinical decisions and resource allocation. Several prognostic scores have been developed to estimate COVID-19 mortality. Among these, the 4C (Coronavirus Clinical Characterisation Consortium) Mortality Score, developed in the United Kingdom, has demonstrated superior predictive accuracy in various settings [1,2]. A recent meta-analysis involving 46,914 patients from multiple countries found that the 4C Mortality Score had the highest pooled discrimination for predicting mortality [1]. Similarly, a systematic review evaluating 1,522 studies and 109 prognostic scores highlighted the 4C Mortality Score as having the largest cohorts and the highest accuracy for mortality prediction [3].

Despite these findings, the performance of prognostic scores is highly dependent on the population to which they are applied [4]. Factors such as age distribution, prevalence of comorbidities, and healthcare system characteristics can significantly influence the accuracy and reliability of these scores. For instance, teaching hospitals in Brazil typically manage a higher proportion of elderly patients and those with multiple comorbidities compared to general hospitals, potentially impacting the applicability of the 4C Mortality Score in this context.

This study aims to address the gap in the literature by evaluating the accuracy of the 4C Mortality Score in predicting in-hospital mortality among COVID-19 patients admitted to a university teaching hospital in Brazil. By examining this score's performance in a high-risk population, we aim to provide evidence for its utility in similar healthcare settings, ultimately contributing to improved patient management during the ongoing pandemic.

## Materials and methods

The study was observational, longitudinal, and retrospective. It was conducted in a 180-bed teaching university hospital in Rio de Janeiro, Brazil. The study included all COVID-19 patients aged 18 or older who were admitted and diagnosed by reverse transcription polymerase chain reaction (RT-PCR) testing of respiratory secretions and/or nasal specimens. Patients discharged in less than 24 hours after admission were excluded. This study was approved by the local ethics committee (approval number: CAAE 48321021.0.0000.5243) and was conducted during the first wave of COVID-19, from March to December 2020. We employed a convenience sampling method, including all eligible patients admitted during the study period.

Clinical, laboratory, and radiologic parameters were collected from the medical records from admission until death or discharge: hemoglobin, leukocytes, platelets, serum urea, creatinine, C-reactive protein (CRP), and D-dimer. The 4C Mortality Score was calculated considering age, sex, Charlson index, respiratory rate, peripheral oxygen saturation (room air), Glasgow Coma Scale, serum urea, and CRP level. The 4C Mortality Score is calculated based on these eight variables, each assigned a weighted score depending on its value or category (Appendix). The total score can range from 0 to 21 points, with higher scores indicating a greater risk of in-hospital mortality. Patients are stratified into four risk categories based on their total score: low risk (0-3 points, <2% mortality), intermediate risk (4-8 points, ~9% mortality), high risk (9-14 points, ~32% mortality), and very high risk (15-21 points, ~62% mortality), providing a clear framework to predict in-hospital mortality for COVID-19 patients [5]. The 4C Mortality Score is a risk stratification tool produced by the International Severe Acute Respiratory and Emerging Infection Consortium (ISARIC) 4C consortium [5]. It is designed for use by clinicians, requiring only parameters commonly available at hospital presentation. While based on a UK cohort of patients, the score should not be adopted for routine clinical use in other settings until it has been appropriately validated.

Additionally, comorbidities, the need for mechanical ventilation, and the use of vasopressors were evaluated, and clinician-defined obesity was established as a comorbidity. Lung involvement in computed tomography (CT) was defined as severe when more than 51% of the pulmonary parenchyma was affected. The primary outcome was mortality, according to the 4C score reference.

The Shapiro-Wilk test was used to evaluate the distribution of data. Results are presented as the median and interquartile range for continuous variables and chi-square statistics for categorical variables. Participants were divided into two groups: discharged and deceased cases. Multivariate Cox regression analysis was performed to identify independent predictors for in-hospital mortality. Explanatory variables included in the multivariate Cox regression were those with a descriptive level lower than 0.20 (p < 0.20) in the bivariate analysis. The variable selection process was stepwise forward, at a significance level of 5%. The accuracy of the 4C Mortality Score was evaluated using the area under the receiver operating characteristic curve (AUC-ROC). Microsoft Excel (Microsoft Corporation, Redmond, Washington) was used for the database, and R software (v.4.1.3, R Foundation for Statistical Computing, Vienna, Austria) was employed for statistical analysis.

## Results

We included 208 patients admitted with COVID-19, of whom 111 (53%) were male, with a median age of 63 years. Table 1 summarizes the baseline clinical and laboratory characteristics. Mortality occurred in 83 (39.9%) of the patients. Compared to discharged patients, those who died were older (67 vs. 59 years, p = 0.005), had significantly lower hemoglobin levels (9.9 vs. 11.9 g/dL, p < 0.001), higher D-dimer levels (2139 vs. 1209 µg/mL, p = 0.021), and elevated CRP levels (14.9 vs. 6.2 mg/dL, p < 0.001).

**Table 1:** Baseline clinical and laboratory features. Continuous data are expressed as the median (p25; p75), and the Mann-Whitney test was performed. Categorical data are presented as n (%), and the chi-squared test was performed. CKD, chronic kidney disease; COPD, chronic obstructive pulmonary disease; CRP, C-reactive protein; CT, computed tomography.

Variables	Discharged (n=125)	Dead (n=83)	p-value
Clinical parameters			
Age (years)	59 (48-70)	67 (62-76)	0.005
Male	66 (53)	45 (54)	0.500
Obesity	19 (15)	14 (17)	0.450
Hypertension	79 (63)	63 (76)	0.480
Diabetes	44 (35)	40 (48)	0.740
Cardiovascular disease	28 (22)	24 (29)	0.760
Malignancy	45 (36)	38 (46)	0.270
Cerebrovascular disease	8 (6)	4 (5)	0.240
CKD	15 (12)	23 (28)	0.100
COPD	20 (16)	20 (24)	0.810
Laboratory baseline findings			
Hemoglobin (g/dL)	11.9 (9.9-13.8)	9.9 (7.9-12.3)	<0.001
Leukocytes (10³/mm³)	8.9 (5.6-13.0)	6.7 (5.2-9.3)	0.490
Platelet count (10³/mm³)	213 (159-273)	208 (143-290)	0.830
Creatinine (mg/dL)	1.01 (0.72-1.25)	1.31 (0.82-2.22)	0.054
D-dimer (mcg/mL)	1209 (597-2861)	2139 (1121-4806)	0.021
CRP (mg/dL)	6.2 (2.9-13.7)	14.9 (7.9-12.3)	<0.001
COVID-19 severity			
Length of hospital stay (days)	14 (2-154)	17 (2-98)	0.159
Mechanical ventilation	13 (10)	66 (80)	<0.001
Hemodynamic instability	16 (13)	68 (82)	<0.001
Severe lung involvement (CT)	30 (24)	47 (57)	0.010

Independent predictors of in-hospital mortality identified in the multivariate analysis are shown in Table 2. These included mechanical ventilation (HR 2.10, 95% CI 1.09-4.04; p = 0.027), hemodynamic instability (HR 2.52, 95% CI 1.27-5.00; p = 0.008), older age (HR 1.02 per year, 95% CI 1.01-1.04; p = 0.007), higher CRP levels (HR 1.02 per mg/dL, 95% CI 1.00-1.03; p = 0.047), and lower hemoglobin levels (HR 0.93 per g/dL, 95% CI 0.86-1.00; p = 0.049).

**Table 2:** Multivariate analysis for independent predictors of mortality. Continuous data expressed as median (p25; p75). Categorical data are presented as n (%). CI, confidence interval; CRP, C-reactive protein; HR, harzard ratio.

Variables	HR (95% CI)	p-value
Mechanical ventilation	2.10 (1.09-4.04)	0.027
Hemodynamic instability	2.52 (1.27-5.00)	0.008
Age (years)	1.02 (1.01-1.04)	0.007
CRP (mg/dL)	1.02 (1.00-1.03)	0.047
Hemoglobin (g/dL)	0.93 (0.86-1.00)	0.049

**Figure 1:**
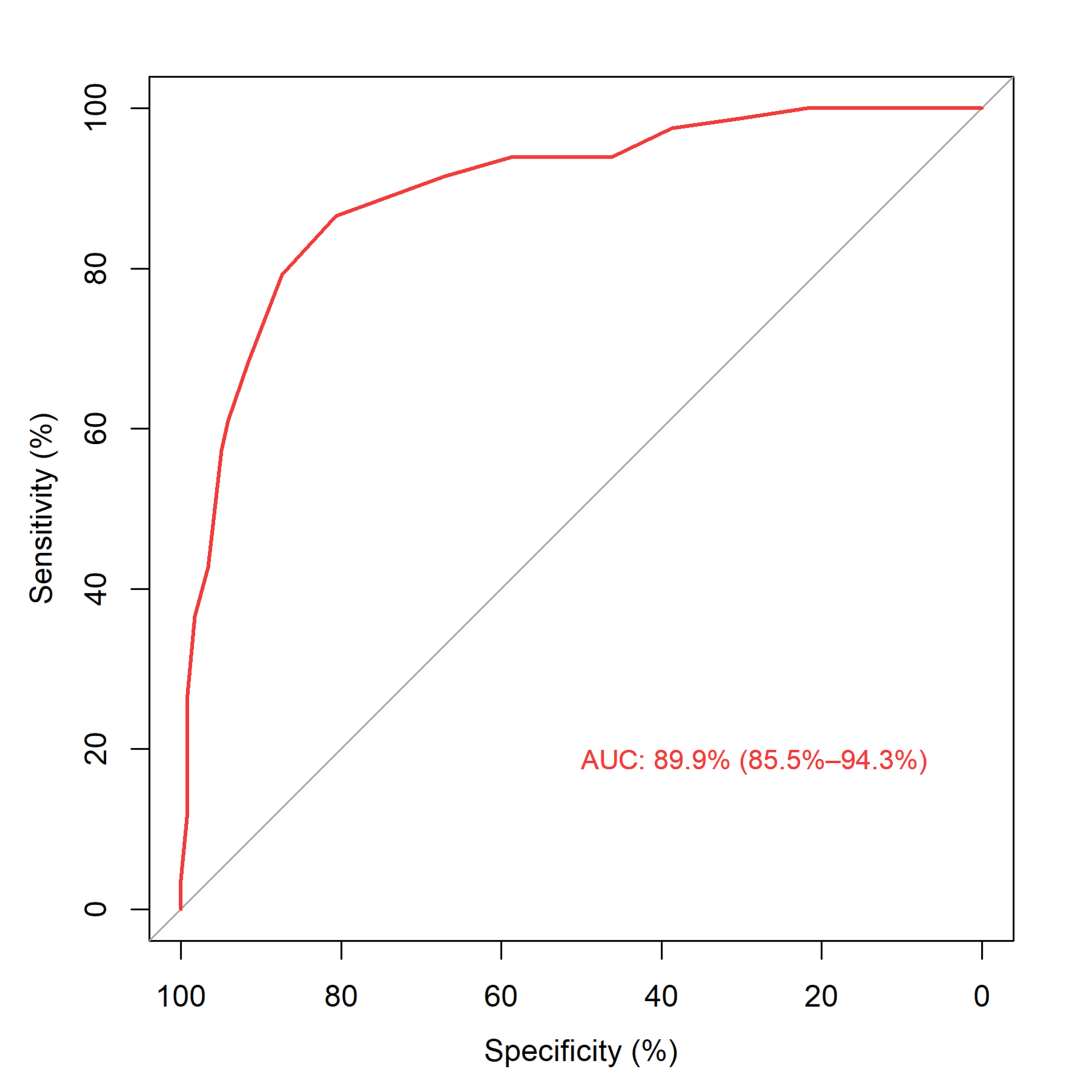
Receiver operating characteristic curve calculated for 4C (Coronavirus Clinical Characterization Consortium) Mortality Risk Score. AUC, area under the curve.

The performance of the 4C Mortality Score was excellent, with an AUC-ROC of 89.9% (95% CI 85.5-94.3%) (Figure 1).

## Discussion

The identification of severe cases of COVID-19 is a fundamental step in selecting appropriate treatment and determining unit allocation. Our study evaluated a simple and practical score, which can be calculated quickly upon admission with minimal laboratory resources and clinical information. Overall, the score performed well, even without tomography results, which may facilitate its application in hospitals and clinics without such technology.

Today, COVID-19 still poses a serious threat to public health, with millions of cases reported each year. The percentage of the population that has been vaccinated remains lower than desired. Consequently, many patients continue to present with severe forms of the disease, with clinical evolution resembling that observed during the first wave [6,7]. For this reason, it is important to have simple and validated tools to identify patients who may develop complications and require more intensive care.

Many scientists have evaluated mortality rates and risk factors in COVID-19 [1-5]. Known predictors of mortality among inpatients include age, obesity, cardiovascular disease, malignancy, inflammatory markers, and pulmonary involvement in CT. Our study demonstrated that hemoglobin levels and hemodynamic conditions are also significant factors to consider when estimating prognosis.

The 4C Mortality Score has been validated in different populations worldwide, with most studies showing good discrimination (AUC-ROC from 0.70 to 0.80) [1-3,5,8]. One study also validated the score for newer COVID-19 variants, such as Omicron [9]. Two previous studies validated the score in Brazil with similar findings, one of which was conducted in a teaching hospital [10,11]. Two recent studies reported better accuracy for the 4C Mortality Score, consistent with our findings [12,13]. This may be because our population included more severe cases of COVID-19. Additionally, our patients were older and had a higher incidence of cardiovascular disease and cancer.

Another possibility is that the teaching hospital where the aforementioned study was conducted operates under an “open doors” emergency policy [9]. Any patient arriving at the hospital can be treated in the emergency room and admitted if necessary. By contrast, our hospital does not offer an "open doors" emergency service. Patient admission is regulated, with sicker patients transferred from other hospitals and emergency services. Additionally, patients with cardiovascular disease and malignancy in our metropolitan area are often referred to our hospital. These factors may account for the differences in populations and results observed in our studies.

Our study has some limitations. The sample size is relatively small, and data were collected from a single institution. However, it is important to note that our hospital serves as a reference for a large metropolitan area with 2 million inhabitants, and we were able to collect data from all patients admitted during the study period. Additionally, our population differs from those in studies conducted at other centers, as our prevalence of cardiovascular disease and malignancy is higher.

## Conclusions

This study evaluated the accuracy of the 4C Mortality Score in predicting outcomes among patients admitted with COVID-19 at a university teaching hospital. The 4C Mortality Score demonstrated excellent discriminatory ability in a population characterized by a higher prevalence of cardiovascular disease and malignancy. These findings underscore the potential utility of the 4C Mortality Score and support the need for future multicenter studies in comparable populations.

## Appendices

**Table 3:** The 4C (Coronavirus Clinical Characterization Consortium) Mortality Score for COVID-19. Comorbidities were high body mass index, cancer, chronic cardiac disease, chronic pulmonary disease, diabetes, liver disease, kidney disease. Altered mental status used Glasgow Coma Scale <15.

Variables	Score
Age (years)	
18-49	0
50-59	+2
60-69	+4
70-79	+6
≥80	+7
Sex	
Female	0
Male	+1
Comorbidities	
0	0
1	+1
≥2	+2
Respiratory rate (breaths/min)	
≤20	0
21-29	+1
≥30	+2
Oxygen saturation, room air (%)	
≥92	0
≤91	+2
Altered mental status	
No	0
Yes	+2
Blood urea nitrogen (mg/dL)	
<20	0
20-39	+1
≥40	+3
C-reactive protein (mg/dL)	
<5.0	0
5.0-9.9	+1
≥10.0	+2

## References

[1] de Jong VM, Rousset RZ, Antonio-Villa NE (2022). Clinical prediction models for mortality in patients with COVID-19: external validation and individual participant data meta-analysis. BMJ.

[2] Monk M, Torres J, Vickery K, Jayaraman G, Sarva ST, Kesavan R (2023). A comparison of ICU mortality scoring systems applied to COVID-19. Cureus.

[3] Appel KS, Geisler R, Maier D, Miljukov O, Hopff SM, Vehreschild JJ (2024). A systematic review of predictor composition, outcomes, risk of bias, and validation of COVID-19 prognostic scores. Clin Infect Dis.

[4] Lombardi Y, Azoyan L, Szychowiak P (2021). External validation of prognostic scores for COVID-19: a multicenter cohort study of patients hospitalized in Greater Paris University Hospitals. Intensive Care Med.

[5] Knight SR, Ho A, Pius R (2020). Risk stratification of patients admitted to hospital with covid-19 using the ISARIC WHO Clinical Characterisation Protocol: development and validation of the 4C Mortality Score. BMJ.

[6] Alshanqeeti S, Szpunar S, Anne P, Saravolatz L, Bhargava A (2024). Epidemiology, clinical features and outcomes of hospitalized patients with COVID-19 by vaccination status: a multicenter historical cohort study. Virol J.

[7] (2021). Clinical characteristics and day-90 outcomes of 4244 critically ill adults with COVID-19: a prospective cohort study. Intensive Care Med.

[8] Gordon AJ, Govindarajan P, Bennett CL, Matheson L, Kohn MA, Camargo C, Kline J (2022). External validation of the 4C Mortality Score for hospitalised patients with COVID-19 in the RECOVER network. BMJ Open.

[9] De Vito A, Colpani A, Saderi L (2023). Is the 4C score still a valid item to predict in-hospital mortality in people with SARS-CoV-2 infections in the Omicron variant era?. Life (Basel).

[10] Costa Mello VL, Americano do Basil PE (2024). Fully independent validation of eleven prognostic scores predicting progression to critically ill condition in hospitalized patients with COVID-19. Braz J Infect Dis.

[11] Avelino-Silva VI, Avelino-Silva TJ, Aliberti MJ (2023). Prediction of intensive care admission and hospital mortality in COVID-19 patients using demographics and baseline laboratory data. Clinics (Sao Paulo).

[12] Zahra A, van Smeden M, Abbink EJ (2024). External validation of six COVID-19 prognostic models for predicting mortality risk in older populations in a hospital, primary care, and nursing home setting. J Clin Epidemiol.

[13] de Santos Castro PÁ, Del Pozo Vegas C, Pinilla Arribas LT (2024). Performance of the 4C and SEIMC scoring systems in predicting mortality from onset to current COVID-19 pandemic in emergency departments. Sci Rep.

